# Supplementary material for: A Global Screen for Assembly State Changes of the Mitotic Proteome by SEC-SWATH-MS
Source: Cell Syst. 2020 Feb 26;10(2):133–155.e6. doi: 10.1016/j.cels.2020.01.001 (PMC7042714; doi:10.1016/j.cels.2020.01.001)

Q9BTC0 | DIDO1_HUMAN | DIDO1 C20orf158 DATF1 KIAA0333
Monomer MW [kDa]: 243.873 Monomer expected elution fraction: 32

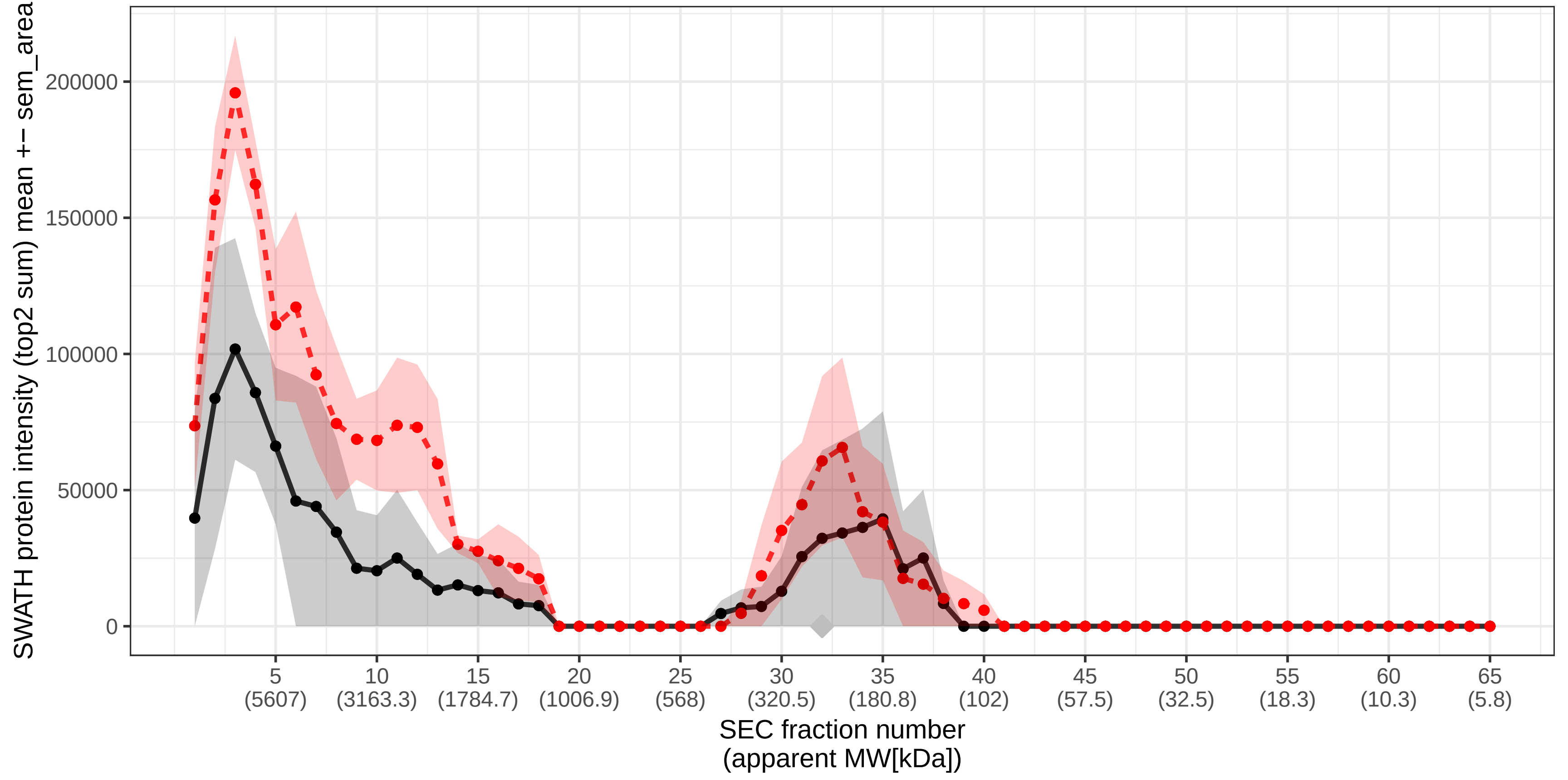

Supplement: Data S1. SEC-SWATH-MS Protein Chromatograms, Related to Figure 1 [file mmc6.zip › SECchrom_Q9BTC0_DIDO1_HUMAN_DIDO1_C20orf158_DATF1_KIAA0333.pdf]
